# Governing Privacy-Preserving Face Recognition in Transport Infrastructures: A Comprehensive Review

**DOI:** 10.3390/s26092832

**Published:** 2026-05-01

**Authors:** Eva María Benito Sanz, Alba Gonzalo Primo, Gaurav Choudhary, Nicola Dragoni

**Affiliations:** Section of Cybersecurity Engineering, Department of Applied Mathematics and Computer Science, Technical University of Denmark (DTU), 2800 Kongens Lyngby, Denmark; s243313@student.dtu.dk (E.M.B.S.); s243343@student.dtu.dk (A.G.P.); gauch@dtu.dk (G.C.)

**Keywords:** face recognition, privacy preservation, airports, public transport, biometrics

## Abstract

Face recognition technologies are increasingly deployed in transport infrastructures to improve efficiency and security, but they raise significant privacy and data protection concerns. This study reviews how privacy-preserving face recognition techniques can address these challenges in real-world settings. Using a systematic literature review approach, the paper analyses research across technical, operational, and governance perspectives. The findings show that while advanced methods such as encryption, federated learning, and de-identification can reduce data exposure, they are rarely implemented in operational systems, which tend to prioritize performance and scalability. At the same time, governance-focused studies emphasize issues such as proportionality, accountability, and fundamental rights, often without clear links to technical solutions. Overall, the review highlights a fragmented landscape and a gap between research and practice, underscoring the need for integrated approaches that align privacy-preserving techniques with practical deployment constraints and regulatory requirements.

## 1. Introduction

Over the past fifteen years, face recognition technology (FRT) has moved from controlled trials to large-scale deployments in airports and public-transport infrastructures worldwide, including Automated Border Control (ABC) e-gates, biometric boarding, seamless-travel initiatives, metro fare gates, and surveillance analytics. In these settings, FRT is typically embedded into end-to-end passenger-processing pipelines (enrolment, identity binding, matching, and exception handling) and integrated with existing security and mobility infrastructures. These systems are promoted as improving throughput, security, and operational efficiency-reducing friction in passenger flows and enabling more automated decision-making but they rely on large-scale acquisition and processing of facial images and biometric templates.

Such deployments raise substantial privacy, data-protection, and fundamental-rights concerns. Facial biometric data are a special category under the EU GDPR, and biometric identification in publicly accessible spaces is classified as high riskunder the EU AI Act [1]. Prior work highlights risks of surveillance expansion, demographic bias, opaque multi-actor data flows, secondary use of biometric templates, and limited alternatives for non-consenting passengers [2,3,4], often exacerbated by centralized architectures and weak governance [5,6]. In transport environments, these risks are amplified by practical compulsion and time pressure: “voluntary” biometric lanes may still shape behavior when opt-out routes are unclear, slower, or socially costly, and errors can translate directly into delays or repeated screening.

In parallel, privacy-preserving face recognition (PPFR) has advanced rapidly. Techniques such as homomorphic encryption, federated learning, reversible de-identification, and synthetic data generation aim to reduce exposure across the recognition pipeline by limiting what is revealed during matching, training, or surveillance processing. Nevertheless, such approaches remain largely absent from operational transport deployments, revealing a persistent research-to-practice gap between what is demonstrated in controlled settings and what is integrated into real-world socio-technical systems.

Motivated by policy discussions and planned deployments in Denmark and across Europe, our exploratory review found that transport is among the most active and contested FRT domains, but the literature remains fragmented across technical, operational, and governance perspectives. This systematic literature review (SLR) addresses three gaps: (i) a lack of transport-specific syntheses connecting PPFR with real deployment and governance conditions [4,7,8]; (ii) limited evidence on adoption barriers in transport (latency, throughput, certification, vendor ecosystems, multi-actor governance); and (iii) a need for up-to-date analysis under expanding AI regulation, particularly the EU AI Act [1].

### 1.1. Contributions

This review makes several key contributions to the study of face recognition (FR) in transport infrastructures. First, it introduces a PRISMA-guided, PICOC-structured systematic literature review protocol that integrates technical, operational, and socio-legal perspectives, ensuring methodological rigor and reproducibility. Second, it provides a comprehensive descriptive analysis of research trends from 2010 to 2025, covering deployment contexts, geographical distribution, architectural patterns, datasets, and evaluation practices.

Third, the review offers a multi-layer comparative synthesis linking PPFR techniques, such as homomorphic encryption (HE), federated learning, secure aggregation, de-identification, to reported transport deployments and regulatory contexts. This synthesis maps privacy and fundamental rights risks to leakage points across the face recognition pipeline, highlighting structural gaps between academic research and operational practice.

Finally, the review develops an integrated socio-technical framework that situates PPFR methods within existing governance and regulatory regimes, including the GDPR, the EU AI Act, and ICAO/IATA standards, and outlines open challenges and future research directions for both technical and policy communities.

### 1.2. Outline

**Figure 1:**
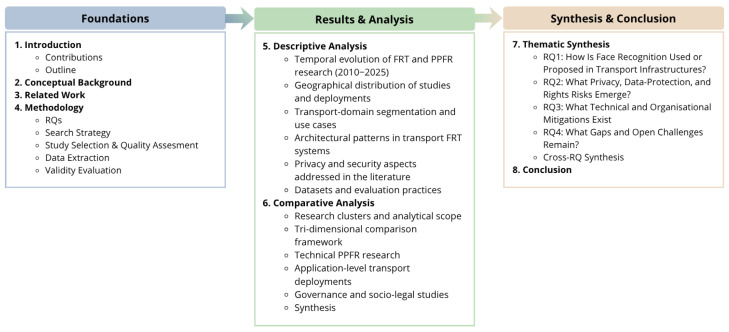
Overview of the paper structure and analytical flow.

The remainder of this paper is organized as follows. Section 2 reviews related work on FR, privacy-preserving biometric systems, and transport security, positioning the present study within the existing body of literature. Section 3 describes the SLR methodology, detailing the research questions, the PRISMA-guided search strategy, the inclusion and exclusion criteria, and the data extraction process. Section 4 presents a descriptive analysis of the selected corpus, examining temporal and geographical trends, application domains, and deployment contexts of FRTs in transport infrastructures. Section 5 provides a comparative analysis of the literature by introducing a tri-dimensional framework that contrasts technical, application-level, and governance-oriented research streams. Section 6 offers a thematic synthesis aligned with the four research questions, identifying key findings, associated risks, proposed mitigations, and open challenges across the reviewed studies. Finally, Section 7 concludes the paper by summarizing the main contributions and outlining directions for future research. The overall structure of the paper is illustrated in Figure 1.

## 2. Conceptual Background

Before proceeding to the related work and review methodology, this section outlines the key concepts and frameworks that form the basis of our systematic literature review. We begin by presenting a conceptual pipeline of face recognition systems (FRS), then discuss the PPFR paradigm, and finally outline the regulatory and governance contexts that influence the deployment of such systems in transport infrastructures.

### 2.1. Face Recognition Systems as a Pipeline

FRS are commonly modeled as a multi-stage pipeline in which raw sensory inputs are progressively transformed into biometric representations that support recognition decisions. This pipeline perspective is well established in the face recognition literature and provides a structured basis for evaluating system performance, security, and privacy risks [9,10].

For transport infrastructures, the pipeline framework allows data flows and associated privacy risks to be mapped systematically across processing stages. Five canonical stages can be distinguished:

1. Acquisition: Raw facial images or video streams are captured from sensors such as CCTV cameras, e-gate kiosks, or turnstile-mounted cameras.
2. Pre-processing: Face detection, alignment, and quality-control filtering are applied to reduce environmental variability (e.g., motion blur, occlusion).
3. Feature extraction: Facial images are transformed into compact biometric embeddings (high-dimensional vectors) by a deep neural network.
4. Template management: Embeddings are stored, indexed, a biometric database for subsequent comparison.
5. Matching and decision: Stored templates are compared against a query embedding in a 1:1 verification or 1:N identification scenario to produce a recognition decision.

Each stage exposes sensitive data artifacts to potential leakage. We adopt a six-level leakage taxonomy (L1–L6) that localizes privacy risks along the pipeline [11]:

- L1: Raw biometrics: Unprocessed frames and face crops at the acquisition stage.
- L2: Intermediate representations:Feature maps and face crops produced during pre-processing.
- L3: Templates and embeddings: Biometric vectors stored in or transmitted to the template database.
- L4: Model parameters: Weights and gradients of the feature extraction model, which can be exploited via inversion or membership-inference attacks.
- L5: Database and metadata: Identity records, query logs, and retention artifacts held in the back-end store.
- L6: Communication channels: Data exchanged across system boundaries, including cross-actor APIs, integration protocols, and audit logs.

**Figure 2:**
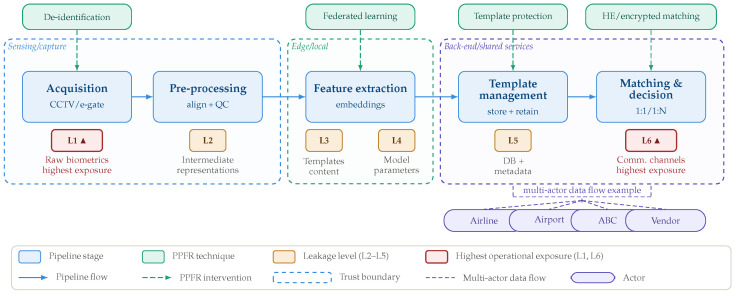
Reference face recognition pipeline for transport infrastructures, highlighting privacy leakage levels (L1–L6), PPFR mitigation techniques, and multi-actor governance complexity.

Figure 2 illustrates how these leakage levels map onto the pipeline stages and trust boundaries, and indicates where PPFR techniques intervene. The figure is read left-to-right: sensing and capture operations (L1–L2) occur at the network edge; feature extraction and template management (L3–L5) are handled by back-end services; and cross-actor integration (L6) spans the full infrastructure. This reference model is used throughout the paper to classify privacy risks and compare mitigation strategies across the reviewed studies.

### 2.2. Privacy-Preserving Face Recognition Paradigm

PPFR encompasses a range of techniques designed to protect sensitive biometric information while maintaining effective recognition performance. These approaches target different stages of the FRS pipeline and collectively aim to reduce the likelihood of sensitive information leakage. For the purposes of this review, we focus on four families of PPFR methods particularly relevant to transport contexts:

- Homomorphic Encryption (HE): Enables computations directly on encrypted biometric data, preventing cleartext exposure during storage and matching operations [12]. HE primarily mitigates risks at L3 and L5.
- Federated Learning (FL): Supports decentralized model training without transferring raw biometric data to a central server, reducing exposure at L1–L2 and limiting centralization risks [13].
- Template Protection (TP): Applies cryptographic or transformation-based methods to secure stored biometric templates against theft or misuse [14], primarily addressing L3.
- De-identification and Anonymization: Alters or suppresses facial data to prevent identification, making these techniques well suited to non-identity analytics such as crowd monitoring or safety surveillance [15]. These methods operate primarily at L1.

No single technique provides end-to-end privacy protection across all pipeline stages. As discussed in the comparative analysis (Section 5), effective mitigation requires combining technical PPFR methods with appropriate architectural choices and governance structures. The leakage taxonomy introduced above (L1–L6) is used throughout the remainder of this review to classify which risks each method addresses and which remain unmitigated in operational transport deployments.

## 3. Related Work: Survey Landscape and Evidence of Fragmentation

Before comparing paper-level evidence, we position our contribution with respect to existing surveys and systematic reviews on Face Recognition (FR) and privacy-preserving biometrics. Table 1 summarizes the analytical scope of representative reviews and highlights which dimensions they systematically cover. The key observation emerging from this comparison is structural fragmentation: most surveys provide depth within a single layer but fail to connect technical mechanisms, deployment contexts, and governance considerations. Technical PPFR surveys focus primarily on cryptographic and template-protection methods [11,14,16], while applied biometric studies emphasize operational constraints and usability in settings such as ABC [7]. Furthermore, governance-oriented works address proportionality and accountability but rarely translate these principles into concrete system architectures or pipeline-level interventions [2,17]. Among existing reviews, Laishram et al. [11] provides a critical methodological reference by explicitly localizing where privacy risks materialize along the face recognition pipeline, and served as the direct source of the L1–L6 taxonomy adopted in this study (Section 2). However, this survey remains primarily technically focused and does not address transport-specific deployment constraints, governance considerations, or the biometric leakage patterns inherent to high-throughput transport infrastructures. As shown in the matrix, no existing review systematically integrates technical, transport-specific, and governance dimensions. This SLR is designed to bridge these disconnected strands by evaluating three core dimensions:

- Technical Leakage Coverage (D1): Identifying where sensitive biometric data are most vulnerable across all pipeline stages using the L1–L6 taxonomy (Section 2), with particular attention to transport-specific exposure patterns.
- Transport Realism (D2): Modeling real-world constraints such as hardware heterogeneity, sub-second latency budgets in biometric gates to avoid queue amplification, and the environmental variability (lighting, motion) of airports and metro stations.
- Governance and Rights Treatment (D3): Explicitly engaging with the lawful basis, necessity, and multi-actor accountability (airlines, authorities, vendors) under frameworks like the **GDPR** and the **EU AI Act**, which classifies biometric identification in public spaces as high-risk.

By addressing these layers simultaneously, this study fills the identified gap in the literature, moving beyond isolated component analysis toward a socio-technical assessment of biometrics in critical transport infrastructures.

## 4. Methodology

This section describes the research method used in this SLR on the deployment of FRTs in airports and public-transport systems. We follow the research method proposed by Petersen in [19] and utilize the suggested structure, including the use of the PRISMA 2020 guidelines method and a PICOC-based scoping strategy to ensure transparency, reproducibility, and methodological rigor.

### 4.1. Research Questions

This SLR examines how FRTs are deployed or proposed in transport infrastructures, with a focus on technical and organizational implementations, associated privacy and fundamental-rights risks, and proposed mitigation strategies. Based on these objectives, the review addresses the following research questions:

- RQ1: How are FRTs deployed or proposed in airport and public-transport infrastructures?
- RQ2: What privacy, data-protection, and fundamental-rights risks are identified in the literature for FRS operating in transport contexts?
- RQ3: What technical and organizational mitigation strategies, including PPFR techniques, are proposed or evaluated for use in transport infrastructures?
- RQ4: What gaps, limitations, and open challenges remain for the deployment of PPFR in transport infrastructures, particularly in light of emerging regulatory frameworks?

Together, these research questions structure the descriptive and comparative analyses and guide the synthesis of risks, mitigations, and open challenges discussed in this review.

### 4.2. Search Strategy

This section describes the search strategy adopted in this study and the methodology used to identify and select the literature included in this SLR. The search process follows established guidelines for systematic literature studies proposed by Petersen et al. [19], incorporates the snowballing procedure described by Wohlin [20], and is informed by prior systematic surveys in security and privacy research (e.g., [21]).

Motivated by these guidelines, search terms were derived from the research questions using a PICOC-based framework, following the approach proposed by Kitchenham and Charters [22]. The PICOC elements were instantiated as follows:

- Population (P): Airports and public-transport infrastructures (e.g., rail, metro, and bus systems), including passengers, operators, and authorities.
- Intervention (I): Deployment or proposal of face recognition systems and PPFR techniques.
- Comparison (C): Conventional face recognition versus privacy-preserving approaches; centralized versus distributed architectures.
- Outcomes (O): Privacy, data-protection, and fundamental-rights risks, together with technical and organizational mitigation strategies.
- Context (C): Real or simulated airport and public-transport environments, including governance and regulatory analyses.

Based on these elements, keywords and their synonyms were identified and combined into search strings using Boolean AND and OR operators. We initially formulated a broad query to capture the general landscape of FRTs in transport infrastructures, including studies that did not explicitly focus on privacy-preserving methods or governance aspects. An example of this initial query is shown below:

Query (Q1)  
("face recognition" OR "biometric identification") AND ("airport" OR "public transport" OR "metro" OR "railway" OR "e-gate")

   This query yielded approximately 2964 studies across the selected databases, providing a broad set of studies. However, the results were largely heterogeneous and did not sufficiently target PPT or specific technical approaches, such as homomorphic encryption (HE) or federated learning, which are central to this review.

Following an initial screening of these results, the search was further refined to target studies explicitly addressing PPFR and related regulatory considerations. The refined query incorporated terms associated with PPFR techniques and data-protection frameworks, while retaining the transport-related keywords:  

Query (Q2)
("face recognition" OR "biometric identification")
AND ("airport" OR "public transport" OR "metro" OR "railway"
   OR "e-gate")
AND ("privacy-preserving" OR "homomorphic encryption"
   OR "federated learning" OR "de-identification"
   OR "template protection" OR "synthetic data")
AND ("privacy" OR "GDPR" OR "data protection" OR "AI Act")

This refined search yielded approximately 523 papers, focusing more specifically on PPFR in transport systems and covering technical, operational, and socio-legal perspectives.

The initial database searches were conducted primarily through Google Scholar and DTUFindIt (https://findit.dtu.dk) which provide comprehensive coverage and full-text access via the DTU account. Subsequently, additional searches were performed across other databases, including IEEE Xplore, ACM Digital Library, Scopus, Web of Science, and SpringerLink, covering publications from January 2012 to June 2026. After the database searches, both backward and forward snowballing were applied to the references and citations of relevant studies.

The detailed inclusion and exclusion criteria, deduplication procedure, and filtering steps are described in Section 4.3.

### 4.3. Study Selection and Quality Assessment

The study selection process follows the PRISMA (Preferred Reporting Items for Systematic Reviews and Meta-Analyses) methodology, ensuring a transparent, systematic, and reproducible screening procedure commonly adopted in SLRs.

The process began with an extensive database search conducted primarily through DTUFindIt using the initial broad Query Q1, which yielded an initial pool of studies. As discussed in Section 4.2, these results largely lacked explicit coverage of complex PP techniques and governance considerations. Consequently, the search was refined using Query Q2, resulting in a more targeted set of papers. As multiple databases were queried, a deduplication step was performed, removing duplicate records before further screening.

Once we had a reasonable amount of initial papers without obvious duplicates, a controlled study selection process was applied using predefined inclusion and exclusion criteria to ensure consistency across reviewers and alignment with the research questions. Studies were included if they met at least one of the following inclusion criteria:

- I1: Studies proposing or evaluating PPFR techniques with potential applicability to transport contexts.
- I2: Socio-legal or governance analyses involving biometric deployments in airports or transport hubs.
- I3: Studies explicitly addressing FR or PPFR systems applied in transport infrastructures (e.g., airports, public transport).
- I4: Studies focusing on privacy, data protection, and governance issues related to biometric deployments in transport contexts.
- I5: Peer-reviewed publications demonstrating sufficient methodological detail to ensure academic rigor.

No restriction was imposed on the number of research questions addressed by a study, as one of the core observations motivating this SLR is that existing works typically focus on isolated aspects rather than covering multiple dimensions simultaneously. Studies were excluded if they met any of the following exclusion criteria:

- E1: Non-English publications.
- E2: Publications dated before 2009.
- E3: Earlier versions of extended works already included in the review.
- E4: Editorials, conference summaries, or methodological guidelines that did not contribute empirical or analytical evidence.
- E5: Non-peer-reviewed materials and non-academic reports that lack sufficient methodological rigor.
- E6: Studies not available in full-text format.
- E7: Books, gray literature, or technical reports.
- E8: Pure FR studies that are not transferable to transport or privacy-preserving contexts, including works evaluated exclusively on generic datasets without domain adaptation or domain-specific validation.
- E9: Purely cryptographic studies without biometric application.

After applying the initial inclusion and exclusion criteria, 181 unique studies remained for further screening. It is important to note that the criteria were initially applied conservatively and subsequently refined as more specific cases were encountered and discussed among the authors.

Next, the remaining papers were evenly divided between both authors, who independently screened the titles and abstracts of the remaining studies to remove irrelevant papers and identify those closely aligned with the research questions. During this stage, 83 studies were excluded, leaving 98 papers for full-text review.

The full-text review was conducted by dividing the papers between the two authors. To mitigate potential bias arising from single-reviewer assessment, several measures were implemented:

- All inclusion and exclusion decisions were explicitly documented.
- A concise analytical description of each study’s key characteristics and relevance was recorded in a shared repository.
- Borderline cases were jointly reviewed and discussed.
- If one author considered a study relevant while the other expressed uncertainty, the study was retained for further evaluation.
- If both authors expressed doubt, or one rejected the study while the other was uncertain, a deeper analysis was performed.

During the full-text screening, 17 papers were excluded due to limited relevance to the research questions or methodological shortcomings. This resulted in 81 studies retained for in-depth analysis. To improve coverage, backward and forward snowballing was then applied to the reference lists and citations of the included studies, yielding 26 additional papers. The final corpus comprised 143 primary studies.

Finally, a quality assessment was conducted independently by both authors to evaluate the robustness of the selected studies and identify potential gaps. Each study was assessed using a five-criterion quality assessment (QA) index, with each criterion scored from 0 to 2 (maximum score of 10):

- QA1: Clarity of research objectives.
- QA2: Methodological transparency.
- QA3: Validity of evaluation methods and datasets.
- QA4: Relevance to transport infrastructures.
- QA5: Explicit consideration of privacy and/or governance aspects.

Studies scoring below 5 were reviewed but not considered primary evidence. Additionally, a post-review process identified 4 papers which, following full-text reading, were deemed relevant to be included in the corpus.

**Figure 3:**
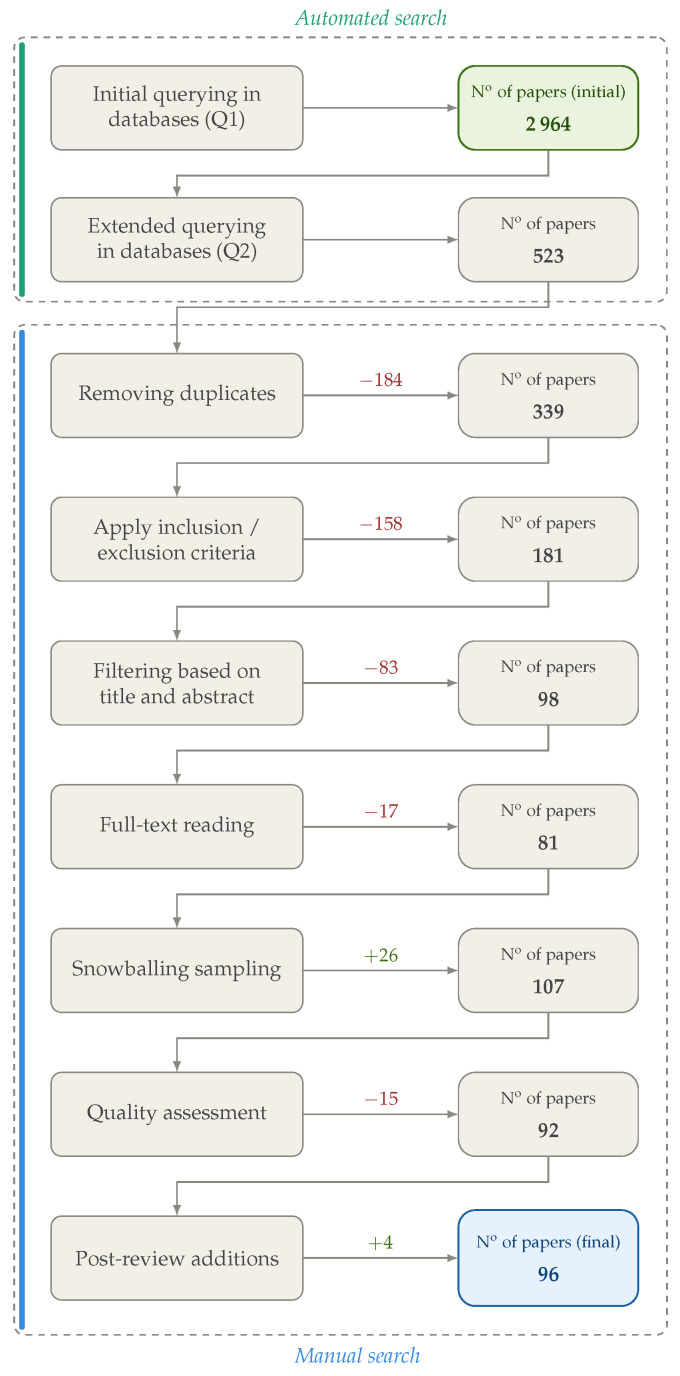
PRISMA flow diagram for study selection.

The entire selection process is summarized in the PRISMA flow diagram shown in Figure 3, which illustrates the number of studies retained and excluded at each stage. By following this structured and transparent process, the final set of studies provides a reliable basis for addressing the research questions while minimizing selection bias.

### 4.4. Data Extraction

To systematically extract data from the selected primary studies, we followed the guidelines proposed by Petersen et al. [19]. Based on these guidelines, a structured data extraction template was defined to ensure consistency, traceability, and comparability across studies. The extracted attributes were selected to capture both the technical characteristics of PPFR systems and the associated governance and regulatory considerations relevant to transport infrastructures.

Data extraction was performed by the second author. To improve reliability and reduce extraction errors, the extracted information was subsequently reviewed by the first author by tracing each entry back to the corresponding statements in the original publications. This validation step, commonly adopted in systematic literature reviews, enhances transparency and supports reproducibility. Table 2 summarizes the data extraction template used in this study.

### 4.5. Validity Evaluation

As with any SLR, the adopted methodology is subject to certain limitations. A primary threat to validity arises from the manual study selection and screening process, which may involve subjective judgment, particularly in an interdisciplinary domain such as FR in transport infrastructures. This risk was mitigated by defining explicit inclusion and exclusion criteria, involving both authors in the screening process, and adopting conservative decision rules whereby studies were retained in cases of doubt.

A further threat concerns potential bias during full-text review and data extraction. To reduce this risk, analytical summaries and exclusion decisions were systematically documented, borderline cases were jointly discussed, and all extracted data were cross-checked against the original studies. In addition, limitations in search coverage due to terminology or indexing were mitigated through the use of multiple databases and backward and forward snowballing.

Finally, to ensure the robustness of the evidence base, a structured quality assessment was applied, and studies below the defined threshold were not treated as primary evidence. Overall, while some threats to validity are inherent to the methodology, the mitigation measures adopted support the reliability and transparency of the review findings.

## 5. Descriptive Analysis

This section presents a descriptive synthesis of the selected studies, aiming to characterize the literature on FR systems in public-transport infrastructures from multiple complementary perspectives, including temporal, geographical, technical, and organizational aspects. Figures and tables are used to support the synthesis and to highlight recurring patterns across the corpus, thereby summarizing the main descriptive findings and establishing the contextual basis for the subsequent comparative analysis.

### 5.1. Temporal Evolution of Face Recognition Technologies and Privacy Preserving Face Recognition Research (2010–2025)

The temporal analysis covers studies published between 2010 and 2025, reflecting the period in which FR systems began to be evaluated and deployed in transport infrastructures, and tracing their evolution towards deep learning–based pipelines, privacy-preserving techniques, and increased regulatory scrutiny. Overall, the literature exhibits a clear progression from early deployment-oriented and performance-driven studies in controlled settings towards a more heterogeneous body of work addressing decentralized learning, privacy-preserving computation, and governance concerns.

In the early period (2010–2014), research relevant to transport infrastructures is largely dominated by ABC and e-gate scenarios in airports. Studies from this phase primarily focus on recognition accuracy, throughput, and usability under controlled conditions, with privacy considerations largely limited to basic template protection. Representative examples include early ABC performance evaluations such as Spreeuwers et al. [23]. At this stage, privacy-related contributions appear mainly as generic biometric protection mechanisms rather than transport-specific solutions.

From the mid-2010s onwards (2014–2020), deep learning has become the dominant paradigm for FR. This shift is reflected in general surveys consolidating advances in deep FR [24,25], as well as in transport-related studies that expand beyond border control to additional infrastructures and sensing contexts. During this period, FR applications begin to appear in scenarios such as in-vehicle monitoring and behavioral or affective analysis (e.g., driver emotion recognition [26]). In parallel, privacy-preserving biometrics gain visibility within the broader biometrics and security communities, with template protection and privacy-enhanced recognition emerging as recurring topics. Early work on cancelable templates and privacy-enhanced recognition established conceptual foundations that later informed PPFR research [27,28].

Between 2020 and 2022, the literature broadens further in terms of deployment scenarios and operational settings. This expansion is partly driven by the increased adoption of computer vision systems in public-transport environments, including monitoring applications in buses, metro entrances, and transport hubs. Representative examples include mask-detection and compliance systems deployed during and post the COVID-19 period [29] and edge-based monitoring solutions for transportation hubs [30]. During the same period, socio-legal and governance-oriented studies gain prominence, addressing issues such as user acceptance, perceived legitimacy, and proportionality of biometric systems in public spaces [31].

From 2022 onwards, PPFR emerges as a central research theme. An increasing number of studies focus on cryptographic protection and secure computation, including homomorphic encryption-based FR and encrypted biometric matching [12,32,33,34]. Federated learning approaches also gain traction, particularly in decentralized and edge-based transport deployments [13,35,36]. In parallel, de-identification and reversible anonymization techniques become increasingly common in transport video analytics and surveillance scenarios [15,37]. Recent work additionally highlights growing attention to evaluation practices and datasets for privacy-aware FR, including synthetic data generation and benchmarking initiatives such as the FRC-Syn challenge [38,39].

**Figure 4:**
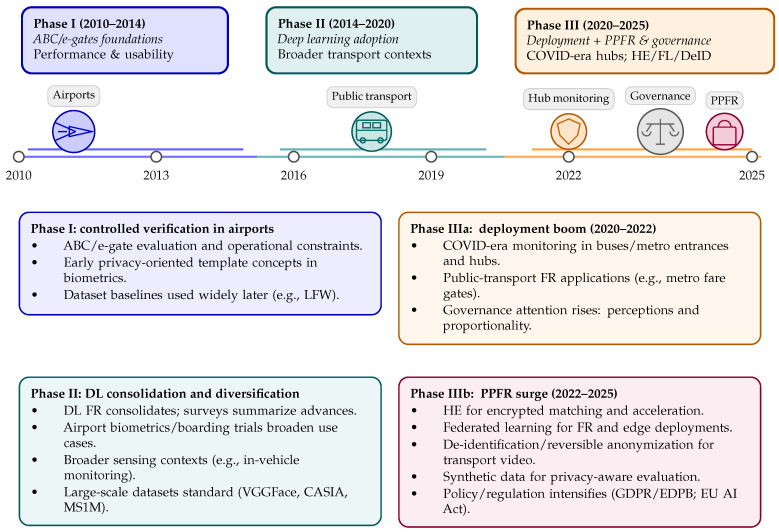
Timeline of FRS and PPFR research relevant to transport infrastructures (2010–2025), highlighting representative milestones across technical advances, deployment contexts, and governance discussions. Supporting references: [1,2,6,10,12,13,15,17,23,24,27,28,29,30,31,32,33,34,35,36,37,38,39,40,41,42,43,44,45,46,47,48,49,50,51].

Figure 4 summarizes this progression by highlighting the main phases in the evolution of FR research relevant to transport infrastructures, from early airport-centric deployments to the recent convergence of large-scale operational systems, privacy-preserving techniques, and regulatory scrutiny.

### 5.2. Geographical Distribution of Studies and Deployments

The selected studies exhibit a pronounced geographical imbalance, reflecting differences in regulatory frameworks, technological capacity, socio-political context, and industry maturity across regions. These differences shape not only where FRS are deployed in transport infrastructures, but also how privacy, governance, and fundamental-rights considerations are addressed in the literature. Based on the corpus analysis, the studies can be grouped into five broad regional clusters.

The Asia-Pacific region dominates the literature on large-scale and operational deployments of FRS in transport infrastructures, particularly in relation to metro systems, airport-wide biometric corridors, smart immigration gates, and national-scale initiatives [29,48]. These deployments typically prioritize performance, throughput, and scalability, often combining FR with IoT-based edge computing and integrated ticketing or identity systems. Privacy and data-protection considerations are generally addressed only marginally or framed primarily in terms of system security.

In contrast, Europe contributes the densest body of socio-legal and governance-oriented research, driven by the GDPR’s classification of biometric data as a special category and reflected in studies on proportionality, legitimacy, and fundamental rights [6,17]. While large-scale operational deployments are less frequently reported than in Asia, European research plays a central role in shaping normative expectations, proportionality assessments, and governance standards for biometric systems in public-transport environments.

North America occupies an intermediate position, combining strong technical and methodological contributions with selected real-world deployments, such as airline biometric boarding pilots and border-control programs, alongside extensive evaluation and fairness studies [52]. Research from this region has been particularly influential in areas such as deep learning architectures, biometric template protection, cryptographic techniques, and fairness auditing. However, the regulatory landscape remains fragmented, with state-level and sector-specific frameworks resulting in less comprehensive biometric governance compared with Europe.

The Middle East and Gulf region is frequently cited for airport-centric biometric deployments, characterized by ambitious implementations aimed at maximizing passenger throughput and seamless travel experiences. These systems are often developed in close collaboration with major industrial vendors (e.g., Dubai Smart Tunnel; biometric boarding at Hamad International Airport). However, academic analysis of governance mechanisms, long-term data management, and privacy-preserving alternatives in this region remains comparatively limited.

**Figure 5:**
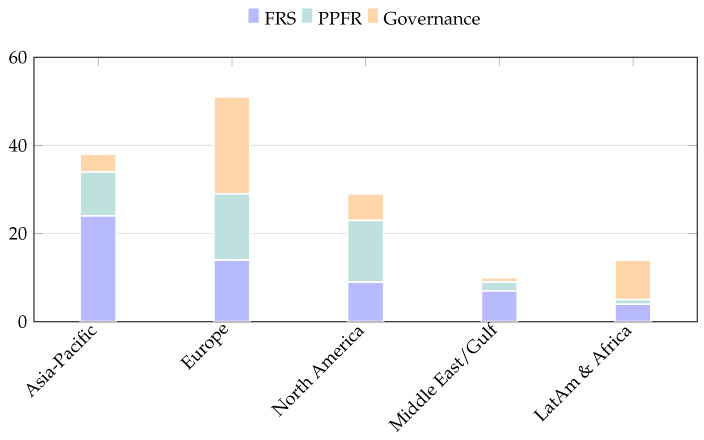
Regional thematic distribution of the selected studies. Bars are stacked by dominant focus: FRS, privacy-preserving face recognition (PPFR), and governance/legal analyses.

Finally, contributions from Latin America and Africa are less prominent in terms of large-scale deployments but are notable for their critical socio-legal perspectives. Studies from these regions frequently examine facial recognition pilots in transport and public spaces through the lenses of accountability, transparency, and the risk of misuse, highlighting concerns related to mass surveillance and institutional safeguards [6]. This body of work provides an important counterbalance to deployment-driven narratives. Figure 5 illustrates these regional differences through a stacked bar chart showing the thematic focus of the selected studies across regions. Each study was assigned to a regional cluster based on its primary deployment context, regulatory focus, or explicitly stated institutional setting, while general surveys and purely methodological contributions without a clear geographical grounding were excluded. Table 3 reports the corresponding counts and provides a complementary quantitative overview of regional research emphases.

### 5.3. Transport-Domain Segmentation and Use Cases

Beyond geographical variation, the selected studies can be systematically segmented according to the transport domain and the concrete operational use cases in which FRS are deployed or analyzed. This distinction is particularly relevant, as different transport domains impose heterogeneous technical constraints, privacy risks, and governance challenges, which in turn shape architectural choices and the feasibility of privacy-preserving techniques.

Moving beyond the traditional dichotomy between ABC and non-ABC applications, the analyzed corpus reveals five distinct transport-domain categories, each characterized by specific operational objectives, data flows, and levels of regulatory and organizational complexity.

The most mature and extensively studied domain remains ABC in airports. Research in this area focuses on controlled identity-verification scenarios such as e-gates, emphasizing recognition accuracy, throughput, and usability under regulated conditions. Studies analyze environmental and operational factors as well as compliance with ICAO and cryptographic requirements [7,8,23,53]. Despite strong regulatory oversight, PPFR techniques are largely absent, with most systems relying on centralized architectures and conventional access-control mechanisms.

Closely related but operationally distinct is biometric boarding, increasingly deployed by airlines and airport operators through industrial platforms [46,48]. Unlike ABC, biometric boarding involves multi-actor data flows across airlines, airports, vendors, and authorities, complicating accountability and governance. While operational benefits are well documented, systematic treatment of data minimization, cross-actor access control, and responsibility allocation remains limited [17,31].

A third prominent domain concerns metro and rail fare gates, where face recognition enables ticketless access control in high-throughput public-transport environments. Studies, predominantly from Asia, prioritize strict latency constraints, robustness to motion and lighting variability, and multi-camera fusion [54]. Privacy considerations are typically implicit, with widespread reliance on centralized storage and limited template protection, reflecting operational priorities and weaker regulatory constraints.

In contrast, station and terminal-level video analytics often decouple face-related processing from explicit identity recognition. Typical applications include mask detection, crowd monitoring, and behavioral analysis in transport hubs [29,30]. These systems frequently adopt edge-computing architectures to reduce data transmission and improve scalability; however, formal privacy threat modeling and systematic leakage analysis remain uncommon. More granular body-level analysis, such as human parsing, offers a complementary non-identifying approach for understanding passenger behavior and crowd dynamics. Recent work has highlighted that such models must remain robust to the image corruptions common in real transport environments, including motion blur, lighting variability, and occlusion [55].

Finally, a smaller but ethically salient category addresses in-vehicle driver monitoring. Studies examine face-based identity verification, emotion recognition, and fatigue detection in buses, trains, and commercial vehicles [26,40,41]. Unlike passenger-facing applications, these systems involve continuous monitoring of workers, raising acute concerns related to consent, power asymmetries, labor rights, and organizational governance.

Table 4 summarizes the key operational constraints, applicable PPFR techniques, and main deployment gaps across the five domains, offering a structured basis for assessing technology-scenario adaptability.

### 5.4. Privacy and Security Aspects Addressed in the Literature

Building on the reference pipeline and leakage taxonomy (L1–L6) introduced in Section 2, this section analyses how privacy and security concerns are addressed across the selected transport-related studies, identifying which leakage levels are explicitly modeled and which mitigation strategies are considered or omitted in operational deployments.

Early transport-focused works, particularly those centered on automated border control, biometric boarding, and metro fare-gate deployments, predominantly expose privacy risks at the lower leakage levels. Raw image leakage (L1) is the most commonly observed issue, especially in systems relying on CCTV archives, e-gate video streams, station surveillance cameras, and in-vehicle monitoring setups [23,29,30]. In many such deployments, raw video data are transmitted to central servers for processing or storage, enabling re-identification, cross-dataset linkage, and long-term surveillance. Mitigation strategies at this level are typically limited to organizational controls or, in more recent studies, to irreversible de-identification, reversible anonymization, or on-device analytics [15,44].

Intermediate feature leakage (L2) is discussed less frequently but remains relevant in deep learning pipelines employing early-fusion or intermediate offloading architectures. Several technical studies demonstrate that intermediate feature representations can be inverted to reconstruct identifiable facial information [56]. However, only a small subset of works explores architectural countermeasures, such as split learning, to reduce this exposure. Within transport deployments, L2 leakage is rarely addressed explicitly, even when edge-backend architectures are adopted.

Template or embedding leakage (L3) emerges as a dominant risk in centralized deployments, including automated border control systems, biometric boarding pipelines, metro fare gates, and cloud-based face recognition services [7,48]. In these contexts, biometric templates are often stored centrally and reused across multiple operational purposes. While template protection techniques such as cancelable biometrics are well established in the broader biometrics literature [28], only a limited number of transport-oriented studies report their practical adoption. Secure matching based on HE or related cryptographic techniques remains largely confined to experimental or research-oriented settings [12,32,49]. In recent research, watermarking into orthogonal moment representations has been proposed as a complementary integrity mechanism [57,58]. At higher levels of abstraction, model leakage (L4) is extensively analyzed in the PPFR and machine learning security literature through attacks such as model inversion, membership inference, and model extraction [59,60]. However, these threats are rarely considered in transport deployments, where commercial systems frequently rely on closed-source models, limited auditing capabilities, and outdated architectures, resulting in weak protection against inference-based attacks.

Database-level leakage (L5) represents a structural risk in multi-actor transport ecosystems. Several studies highlight that biometric databases are frequently shared across airlines, airport operators, border authorities, municipal transport agencies, and cloud vendors [17,31]. Despite this complexity, few works provide detailed accounts of access-control mechanisms, encryption-at-rest policies, retention limits, or enforcement of purpose limitation, leaving database governance largely opaque.

Finally, protocol-level leakage (L6) remains one of the least explored yet potentially most consequential aspects of transport infrastructures. Interactions between heterogeneous systems, such as airline platforms, airport IT systems, border-control databases, and third-party biometric vendors, often rely on proprietary or undocumented interfaces [2,6]. These protocols may leak metadata, identity flags, query logs, or temporal information that can be exploited to reconstruct passenger mobility patterns. Despite its relevance, protocol-level leakage is seldom modeled explicitly in the PPFR literature, revealing a persistent gap between algorithm-level cryptographic protections and real-world deployment architectures.

### 5.5. Architectural Patterns in Transport Face Recognition Technology Systems

The analysis of the selected studies reveals a limited set of recurring architectural patterns for FRS deployed in transport infrastructures. These patterns are primarily shaped by operational constraints, such as throughput, latency, and deployment scale, as well as by the organizational and regulatory context. Across the corpus, four dominant architectural paradigms can be identified.

The most prevalent pattern is the centralized backend architecture, particularly common in ABC, biometric boarding, and metro fare-gate deployments. In this configuration, biometric capture occurs at distributed checkpoints, while template storage, matching, and decision-making are handled centrally. Studies adopting this architecture typically emphasize performance, reliability, and integration with existing identity infrastructures, such as national border systems or airline databases [7,23]. Centralized designs dominate early airport deployments and large-scale transport systems, where operational simplicity and unified management are prioritized.

A second recurring pattern is the hybrid edge-backend architecture, which has become increasingly prominent in more recent deployments. Here, face detection, alignment, and feature extraction are performed at the edge, while identity matching or resolution remains backend-based. This approach is reported in metro and rail fare gates as well as in modern airport infrastructures seeking to reduce raw video transmission and meet strict real-time constraints [30]. Although this pattern improves data minimization compared with fully centralized designs, biometric templates are still typically managed at the backend level.

A third category comprises edge-only analytics architectures, mainly applied to non-identity-related use cases such as mask detection, crowd monitoring, behavior analysis, or safety surveillance. In these systems, all processing is performed locally on edge devices deployed in stations or vehicles, and only aggregated or anonymized outputs are retained or transmitted [29,30]. This pattern aligns strongly with data minimization principles and is common in monitoring-oriented applications rather than identity verification.

Finally, a smaller but growing body of work explores cloud-assisted secure and privacy-preserving architectures. These designs, largely investigated within PPFR research, rely on techniques such as HE, secure multi-party computation, trusted execution environments, or federated learning to limit exposure of biometric data during matching or training [12,13,32,33,34,49,61]. Despite increasing academic interest, such architectures are predominantly evaluated in experimental or pilot settings and remain rare in large-scale operational transport deployments.

**Figure 6:**
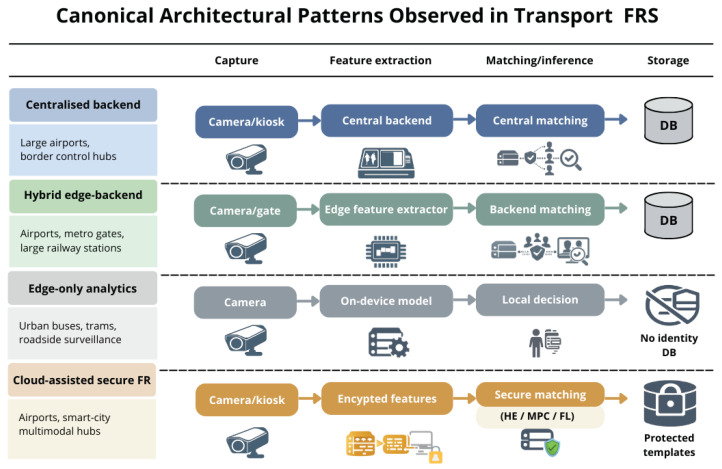
Canonical architectural patterns observed in FRS deployed in transport infrastructures.

Figure 6 synthesizes these canonical architectural patterns, illustrating how FRS are commonly structured across the stages of the adopted baseline FR pipeline.

### 5.6. Datasets and Evaluation Practices

This subsection characterizes the datasets and evaluation practices reported in the selected studies, focusing on how FRS in transport contexts are empirically assessed. Rather than comparing performance outcomes, the objective is to describe dataset provenance, representativeness, and dominant evaluation methodologies across the literature.

Across the corpus, evaluation practices are largely inherited from the broader FR community and rely predominantly on established public benchmark datasets, including Labeled Faces in the Wild (LFW) [45], CASIA-WebFace [47], *VGGFace* [24], and MS-Celeb-1M [25]. These datasets are commonly used for training or pre-training models later applied to transport scenarios. While they support comparability and reproducibility, they are collected under controlled or semi-controlled conditions and fail to capture key characteristics of transport environments, such as motion blur, non-frontal poses, occlusions, and heterogeneous sensing conditions. As a result, their representativeness for real-world transport deployments remains limited, a concern also highlighted in large-scale benchmarking efforts such as the NIST Face Recognition Vendor Test (FRVT).

Only a small subset of studies evaluate FRS using transport-domain or operational datasets collected in real deployment settings, including airports, metro stations, fare gates, buses, or in-vehicle environments. Reported examples include metro and turnstile datasets from Asia, airport-specific masked-face datasets introduced during the COVID-19 period, and proprietary industrial datasets associated with biometric boarding or automated border control systems. However, most such datasets remain closed or proprietary, limiting transparency, reproducibility, and cross-study comparability.

In response to data availability and privacy constraints, synthetic and simulated datasets have gained increasing attention, particularly in PPFR-oriented and governance-aware research. Synthetic face datasets generated using 3D morphable models or generative adversarial networks, as well as benchmarking initiatives such as the FRC-Syn challenge [38,39], are used to enable evaluation without exposing real biometric data. These datasets are typically positioned as complementary resources rather than substitutes for real-world transport data.

Evaluation protocols remain largely centered on classical biometric metrics, including verification accuracy, false acceptance rate (FAR), false rejection rate (FRR), and equal error rate (EER). Deployment-oriented studies occasionally report additional operational metrics such as latency, throughput, or robustness under variable illumination and motion conditions. By contrast, explicit and standardized evaluation of privacy risks, leakage levels, or regulatory compliance remains rare and is usually addressed qualitatively rather than through standardized metrics.

Table 5 summarizes the main dataset categories and their limitations in transport-related FRS studies.

## 6. Comparative Analysis

This section develops the comparative contribution of our SLR. While the descriptive analysis (Section 5) maps what the literature covers, the comparative analysis explains Why different strands of work produce evidence that is difficult to translate across communities. Concretely, we identify structural differences in how distinct research clusters conceptualize (i) privacy risk, (ii) operational feasibility in transport infrastructure, and (iii) governance and rights constraints.

Two recurring tensions motivate the comparative lens. First, technical work on PPFR typically evaluates leakage/security properties under controlled assumptions (e.g., encrypted matching or inference attacks), whereas application-level deployment work is driven by throughput, latency, robustness, and integration constraints in real infrastructures [7,11,16,23]. Second, governance and surveillance scholarship frames the problem as one of legitimacy and fundamental rights (necessity, proportionality, discrimination), where strong technical protections do not automatically imply lawful or socially acceptable deployment [2,3,4,17]. These tensions shape what is measured, what is reported, and what becomes deployable.

### 6.1. Research Clusters and Analytical Scope

Across the corpus, we identify three analytically distinct but partially overlapping clusters. We foreground them because most “missing” connections in the literature arise at their boundaries.

(C1) Technical PPFR research. This cluster proposes cryptographic, distributed, or transformation-based methods that reduce information leakage at specific stages of the FR pipeline (e.g., template protection, encrypted matching, or privacy-preserving training) [11,16]. Evaluation is typically benchmark-driven, and success is measured through security arguments and recognition utility (accuracy/overhead), with limited transport-domain realism.

(C2) Application-level transport deployments. This cluster documents or evaluates operational systems in airports (ABC and boarding), metro/rail gates, transport hubs, or vehicles. Dominant metrics are throughput, latency, FAR/FRR, and robustness under environmental variability [23,48]. Privacy is often treated as “handled elsewhere” (compliance, procurement, or organizational policy) rather than as a design variable; technical safeguards (e.g., encrypted matching or revocable templates) are seldom described in enough detail for independent assessment.

(C3) Governance, socio-legal, and surveillance studies. These works treat FR in mobility spaces as a question of legitimacy, accountability, and fundamental rights. They operationalize necessity and proportionality reasoning and foreground differential impacts, transparency/agency, and power asymmetries [2,3,4,17,51,62]. Technical feasibility is rarely modeled, but governance criteria are often more concrete than in deployment papers (e.g., requirements on alternatives, retention, documentation, and audits).

To provide a compact snapshot before the detailed comparison, Table 6 summarizes how each cluster typically performs across our three comparative dimensions (D1–D3).

### 6.2. Tri-Dimensional Comparison Framework

To compare evidence across clusters without collapsing their different assumptions, we use three jointly necessary dimensions.

D1: Technical leakage coverage. Which artifacts are protected or exposed at which pipeline stage? We operationalize this using the L1–L6 leakage taxonomy, distinguishing capture (L1), intermediate representations (L2), templates/embeddings (L3), model parameters (L4), databases/metadata (L5), and communications/integration (L6). This abstraction is consistent with PPFR threat models and makes deployment architectures comparable to security claims [11,59,63,64].

D2: Deployment realism. Does a study model transport constraints (queue dynamics, latency budgets, hardware heterogeneity, environmental variability, and multi-actor integration)? Gate systems routinely require sub-second end-to-end interactions to avoid queue amplification, which constrains the feasibility of heavier PPFR approaches [7,8].

D3: Governance and rights treatment. Does the work engage with lawful basis, necessity/proportionality, transparency/agency, accountability across actors, and fairness/discrimination obligations? EU data protection law (GDPR), DPA guidance, and the AI Act provide operational criteria that are often absent from technical and deployment papers [1,2,51,52,62].

### 6.3. Tri-Layer Misalignment: PPFR vs. Deployments vs. Governance

A central comparative finding is a persistent tri-layer misalignment: each cluster optimizes a different objective function and therefore produces evidence that is not directly portable to the others. Table 7 grounds this claim with exemplar references per layer.

This misalignment explains why strong PPFR results (e.g., HE/MPC confidentiality) often fail to appear in deployed transport systems, why deployments can be operationally viable but governance-opaque, and why governance requirements can be normatively robust but technically underspecified. The subsections below unpack this misalignment at the paper level using D1–D3.

### 6.4. Comparative Analysis of Technical Privacy Preserving Face Recognition Research

Technical PPFR constitutes the largest methodological cluster in the corpus. To make its internal diversity comparable, we organize PPFR by what artifact it protects (D1) and by what feasibility assumptions it makes (D2), and then indicate which governance questions are typically left implicit (D3).

#### 6.4.1. Cryptographic Matching (HE/MPC): Strong Secrecy, Weak Gate-Fit Unless Optimized

Encrypted matching protects templates/embeddings (L3) and, in some designs, parts of the model logic (L4–L5), preventing the server from learning the queried biometric [12,27,32,49,61,67,68,69,70,71,72,73]. However, many works assume stable, powerful servers and do not model end-to-end gate timing. Transport deployments are latency- and queue-sensitive; direct transfer is difficult without hardware-aware optimization, careful key management, and certified architectures [7,8]. Critically, cryptographic PPFR threat models typically abstract away multi-actor responsibility (airport vs airline vs border authority vs vendor), even though this division of roles is central to accountability under data protection law [2,65].

#### 6.4.2. Template Protection (BTP/Cancelable Biometrics): Revocability with Modern Inversion Caveats

Cancelable templates and broader biometric template protection provide revocability and diversity at L3, aiming to reduce harm after compromise [14,28,74]. Comparative evidence shows that evaluation protocols and adversarial assumptions vary widely; bridging to modern inversion and linkage threats remains an active theme and is underreported in transport-oriented studies [11,14]. Orthogonal moment-based watermarking represents an emerging variant [57], though recent diffusion-based removal demonstrates that robustness against conventional attacks does not hold under adversarial conditions [58]. From a governance standpoint, revocability can support minimization and proportionality arguments only if retention and purpose limitation are enforced in deployments [17,62].

#### 6.4.3. Trusted Execution Environments (TEEs): Operationally Plausible, Governance-Heavy

TEE-based approaches isolate sensitive inference or matching in enclaves [66,75,76]. Compared to HE/MPC, TEEs can better match gate and kiosk constraints (D2), but shift the trust boundary to hardware vendors and introduce patching and attestation governance questions that must be specified for credible assurance [77,78,79]. In transport infrastructures, these questions are practical: multi-actor responsibility for enclave provisioning, key custody, and incident response must be documented to meet accountability standards [2,51].

#### 6.4.4. Federated and Distributed Learning: Reduces Raw-Data Centralization but Does Not Eliminate Leakage

Federated learning (FL) is often motivated as “data stays local” (L1–L2), aligning with distributed transport operators or station-level silos [35,36,42]. Yet gradients and updates can leak membership or reconstruct examples unless additional protections are used [64,80,81]. In transport, non-IID conditions across stations or airlines can degrade utility and exacerbate demographic disparities, so FL requires both security add-ons (secure aggregation/DP) and fairness-aware evaluation [52,82].

#### 6.4.5. De-Identification and Synthetic Data: Best Fit for Analytics and Lifecycle Governance

De-identification targets early pipeline stages (L1) and is most compatible with non-identifying analytics in stations/terminals rather than identity verification [15,43,44,83,84,85,86,87]. Synthetic data addresses governance of training corpora (consent, demographic balance, coverage) without changing runtime leakage directly [38,39,88]. These approaches create a clearer bridge to governance criteria (minimization, purpose limitation), but require explicit reporting of re-identification risk, data provenance, and deployment boundaries to be credible to DPAs [2,62].

#### 6.4.6. Paper-Level Evidence PPFR

Table 8 makes these trade-offs explicit at the paper level. A consistent pattern is that many PPFR papers focus on protecting L3–L5, while L1/L2 and L6 receive less systematic attention, even though transport deployments routinely expose L1 and L6 through pervasive capture and vendor/cloud integration.

### 6.5. Comparative Analysis of Application-Level Transport Deployments

Application-level studies are comparatively strong on D2 (realistic constraints) but weak on D1 (explicit leakage/threat mapping) and especially weak on D3 (reported governance safeguards). We compare deployments through their architectural patterns and the evidence they report.

#### 6.5.1. Architectural Convergence: Centralized or Hybrid Matching Dominates

Across ABC, boarding, and metro gate systems, a common pattern is captured at the edge (L1), local quality and liveness checks, and centralized matching against a database (L3/L5), often mediated by vendors or cross-organization integration (L6) [46,91]. This architecture is operationally convenient, but expands the attack surface and amplifies governance burdens (documentation, roles, retention, transfers).

#### 6.5.2. Reporting Gap: Privacy and Fairness Are Rarely Operationalized

Even when deployment papers acknowledge privacy concerns, they rarely specify retention periods, lawful basis, opt-out procedures, or audit mechanisms, limiting independent assessment [2,62]. Similarly, subgroup performance reporting is uncommon despite benchmark evidence that demographic error disparities can produce unequal treatment in gate-based systems [52]. Operationally, fairness gaps are not abstract: a higher FRR for a demographic group results in longer queues, secondary screening, or repeated manual checks.

#### 6.5.3. Non-Identifying Analytics as a Partial Exception

Video analytics in hubs (e.g., mask/distance monitoring, crowd safety) more often uses edge-only processing and can be compatible with de-identification because identification is not essential to the task [29,30,92]. However, formal threat models and re-identification evaluation are still rare.

#### 6.5.4. Paper-Level Evidence Application-Level Transport Deployments

Table 9 summarizes representative deployments and highlights the recurrent pattern: exposed L1/L3/L5/L6 artifacts with sparse reporting of governance safeguards.

### 6.6. Comparative Analysis of Governance and Socio-Legal Studies

Governance sources contribute two kinds of comparative evidence that are largely missing elsewhere.

First, they provide operational criteria for legitimacy: necessity and proportionality tests require demonstrating that the same objective cannot be achieved with less intrusive means, and that meaningful alternatives exist [2,17,62]. This directly challenges an implicit assumption in some technical work: that stronger privacy controls automatically imply acceptability.

Second, governance sources foreground multi-actor accountability. Airports, airlines, border agencies, transport operators, and vendors jointly shape data flows; yet this is exactly where technical threat models and deployment studies are least explicit [46,65]. As a result, even when a system could be made technically safer (e.g., with TEEs or encrypted matching), responsibilities for key management, retention, incident response, and oversight remain unclear.

Table 10 maps transport-relevant governance sources to the concrete questions they operationalize and how we use them as comparative criteria.

### 6.7. Synthesis: What the Comparison Explains (And What It Enables Next)

Across clusters, three synthesis points follow directly from the evidence.

(S1) Leakage and feasibility trade-offs are systematic. HE/MPC approaches are strongest on confidentiality but often weakest on gate-fit; TEEs are more deployable but raise supply-chain and accountability requirements; de-identification fits analytics but not identity verification. These patterns are stable at paper level (Table 8).

(S2) Transport deployments concentrate exposure at L1 and L6. Pervasive capture and vendor/cloud integration mean that the earliest and latest pipeline stages are routinely exposed, yet are comparatively under-protected in PPFR designs and under-described in deployment reporting.

(S3) Governance criteria define acceptability, not only compliance. DPA guidance and proportionality reasoning require evidence of alternatives, agency, and accountability that cannot be inferred from technical results alone. This is why the tri-layer misalignment persists (Table 7).

These synthesis points set up the thematic synthesis and research agenda in the next section, where we align comparative findings with the project research questions introduced in Section 7.

## 7. Thematic Synthesis

This section integrates the descriptive findings (Section 5) and the comparative analysis (Section 6) into a unified thematic synthesis structured around RQ1–RQ4 (Section 4.1). We follow a hybrid inductive–deductive approach: evidence is mapped to each RQ, while cross-cutting socio-technical patterns are abstracted across clusters. Two patterns recur throughout: infrastructural opacity (unclear roles and data flows) and governance debt (limited reporting of concrete safeguards), both of which contribute to a persistent technical–legal gap between what systems can guarantee technically and what deployments must justify normatively and legally [2,51].

### 7.1. RQ1: How Are FRT Deployed or Proposed in Airport and Public-Transport Infrastructures?

Across the corpus, FRT deployments and proposals concentrate in recurring transport settings: (i) ABC e-gates, (ii) biometric boarding, (iii) metro/rail fare gates, (iv) station/terminal video analytics, and (v) in-vehicle monitoring. While the underlying pipelines vary, the same operational constraint dominates: these systems must work reliably under high throughput and tight time budgets, which tends to privilege architectures that are straightforward to integrate and operate at scale [7,48].

#### 7.1.1. Theme RQ1-A: Throughput-Driven Identity Verification

Gate-based uses (ABC and fare control) treat face recognition primarily as a rapid identity verification primitive. This encourages designs that minimize per-transaction delay and favor stable operational performance, sometimes at the expense of explicit privacy-by-design choices [4,23].

#### 7.1.2. Theme RQ1-B: FRT Is Embedded in Wider Sensing Ecosystems

FRT components are typically embedded within broader smart-transport stacks (multi-camera sensing, edge devices, and passenger-flow or safety analytics). This integration increases the number of interfaces and expands the set of actors who may access, process, or influence biometric processing [30].

#### 7.1.3. Theme RQ1-C: Vendor-Led Architectures Shape What Is Feasible

In many operational contexts, procurement and vendor platforms strongly influence system design and upgrade cycles. As a result, deploying novel PPFR techniques often faces non-technical barriers: integration friction, certification, and dependency on proprietary components [46].

### 7.2. RQ2: What Privacy, Data-Protection, and Fundamental-Rights Risks Are Identified in the Literature for FRS Operating in Transport Contexts?

The literature converges on a small set of recurring risk clusters that are intensified by the transport setting (high stakes, time pressure, and constrained user choice).

#### 7.2.1. Theme RQ2-A: Surveillance Expansion and Function Creep

Because transport infrastructures are highly instrumented and users often cannot practically avoid them, FRT can enable ambient surveillance and later expansion to new purposes (e.g., security, policing, commercial uses), raising proportionality and legitimacy concerns [2,3].

#### 7.2.2. Theme RQ2-B: Infrastructural Opacity in Multi-Actor Data Flows

A recurrent problem is the lack of clarity over who stores templates/embeddings, who performs matching, where processing occurs, and how long artifacts are retained. This opacity makes it difficult to assess compliance, assign responsibility, or enable meaningful oversight [51,62].

#### 7.2.3. Theme RQ2-C: Under-Modeled Leakage and Attack Surfaces

Technical work shows that models and interfaces can leak sensitive information (e.g., membership inference or inversion), but operational accounts often do not translate these threat models into deployment documentation and controls, creating a mismatch between assumed and actual exposure [56,59].

#### 7.2.4. Theme RQ2-D: Fairness and Performance Disparities Under Transport Conditions

Transport conditions (lighting variability, motion blur, occlusion) can amplify error rates, and demographic differentials observed in evaluations can translate into unequal burdens (e.g., repeated checks, delays, exclusion from “fast lanes”). Yet many deployment descriptions do not provide consistent subgroup reporting or auditing detail [52,94].

#### 7.2.5. Theme RQ2-E: Constrained Agency and Weak Consent

Governance sources emphasize that meaningful consent and effective alternatives are difficult to realize in “must-move” environments, particularly when opt-out routes are unclear, slower, or socially costly [2,62].

### 7.3. RQ3: What Technical and Organizational Mitigation Strategies, Including PPFR Techniques, Are Proposed or Evaluated for Use in Transport Infrastructures?

Mitigation strategies fall into two interacting layers: technical PPFR mechanisms and organizational/governance safeguards. A consistent finding is that effective mitigation is socio-technical: technical protections reduce certain exposures, but governance determines whether systems remain accountable and contestable in practice.

#### 7.3.1. Theme RQ3-A: PPFR Reduces Specific Leakages but Is Not Sufficient Alone

The PPFR literature proposes cryptographic matching (HE/FHE), trusted execution for protected inference, and template protection as mechanisms to reduce leakage at key points in the pipeline. These approaches can strengthen confidentiality, but do not by themselves establish proportionality, transparency, or effective redress [61,76,95,96].

#### 7.3.2. Theme RQ3-B: Organizational Safeguards Are Central, but Weakly Evidenced in Deployments

Data minimization, retention limits, access control and logging, and meaningful alternatives are repeatedly highlighted as core safeguards. However, transport deployments are often described primarily in terms of operational performance, with limited detail on how these governance measures are implemented end-to-end [51,62].

#### 7.3.3. Theme RQ3-C: Regulation Creates Design Constraints Rarely Modeled Technically

Emerging high-risk governance expectations require documentation, oversight, and risk management that must be engineered into socio-technical architectures rather than appended after deployment. This includes traceability, accountability mechanisms, and demonstrable controls appropriate to context [1,2].

### 7.4. RQ4: What Gaps, Limitations, and Open Challenges Remain for the Deployment of PPFR in Transport Infrastructures, Particularly in Light of Emerging Regulatory Frameworks?

Five persistent gaps remain, and several are sharpened by tightening regulatory expectations.

#### 7.4.1. Gap 1: Translation Gap from PPFR Research to Deployed Transport Systems

Even when PPFR is technically feasible, latency budgets, integration complexity, and vendor dependence can block adoption in operational environments [46,67].

#### 7.4.2. Gap 2: Missing Multi-Actor Governance Models Compatible with PPFR

End-to-end responsibility allocation (e.g., key control, auditability, retention, redress) is seldom specified, yet it is central for demonstrating accountability and compliance in complex vendor/operator ecosystems [51,62].

#### 7.4.3. Gap 3: Limited Transport-Specific Fairness and Robustness Benchmarks

Benchmarking and evaluation practices often under-capture transport stressors and the operational consequences of subgroup errors, limiting the evidence base for “fair” deployment claims [52].

#### 7.4.4. Gap 4: PPFR Evaluations Under-Model Real Deployment Conditions

Many PPFR evaluations remain laboratory-centric, omitting end-to-end timing, heterogeneous hardware, and operational dynamics that dominate transport feasibility [16].

#### 7.4.5. Gap 5: Under-Studied Protocol-Level Transparency and Engineered Alternatives

Interface-layer risks (APIs, logs, metadata, cross-actor linkages) and the practical engineering of opt-out routes remain under-analyzed, despite being central for transparency and contestability under modern governance expectations [2,51].

### 7.5. Cross-RQ Synthesis

Across RQ1-RQ4, a structural misalignment persists between (i) PPFR research focused on leakage reduction, (ii) deployments optimized for throughput and integration, and (iii) governance frameworks centered on rights and accountability [1,46,51]. Taken together, these findings indicate that the slow translation of PPFR into operational transport systems is primarily structural, which frames the conclusions and research agenda in Section 8.

## 8. Conclusions

This SLR examined how FRT are deployed or proposed in transport infrastructures, where privacy, data-protection, and fundamental-rights risks are identified, what technical and organizational mitigations (including PPFR) are evaluated, and what gaps remain under emerging regulatory frameworks (RQ1-RQ4). Synthesizing evidence across technical, deployment, and governance literature yields three overarching conclusions.

First, transport deployments are predominantly shaped by operational requirements, throughput, reliability, and integration with existing vendor ecosystems, which drives architectural convergence toward centralized or hybrid processing and limits the practical adoption of PPFR techniques in real-world systems [31,46,92]. Second, the transport context amplifies risks that are not purely technical: constrained user agency, multi-actor opacity, and surveillance expansion interact with documented technical attack surfaces and demographic disparities, creating rights impacts that are difficult to mitigate through technical controls alone [2,51,52,62]. Third, while PPFR research provides a rich toolbox, including cryptographic matching, trusted execution, template protection, and privacy-enhancing learning, most evaluations remain laboratory-centric and insufficiently connected to end-to-end operational constraints and governance requirements typical of transport infrastructures [16,61,76,95,96].

Taken together, these findings reveal a persistent structural gap between: (i) PPFR research focused on leakage reduction under formal threat models, (ii) transport deployments optimized for high-throughput service delivery, and (iii) governance frameworks centered on legality, accountability, transparency, and contestability [1,2,51]. Closing this gap requires treating PPFR as a socio-technical design problem rather than a drop-in cryptographic upgrade. Concretely, future work should prioritize: (1) end-to-end PPFR pilots under realistic transport constraints (latency budgets, heterogeneous hardware, multi-camera pipelines); (2) explicit multi-actor governance and accountability models aligned with DPIA-style practice (roles, key control, auditing, retention, and redress); and (3) transport-specific evaluation protocols that integrate robustness and fairness reporting with operational impacts (e.g., queuing effects, secondary screening, exclusion pathways) [52,62,94].

Finally, the rapid evolution of regulatory expectations for biometric and other high-risk AI systems increases the importance of transparent documentation, meaningful alternatives, and demonstrable risk management in transport deployments [1,2]. Overall, the corpus suggests that progress will depend less on any single technical breakthrough than on integrated architectures that jointly satisfy operational feasibility, privacy preservation, and fundamental-rights compliance.

## Figures and Tables

**Table 1:** Survey-level coverage matrix. ✓ = covered; ∼ = partial/implicit; ✗ = not covered.

Survey	Year	L1–L6	HE/MPC	TEE	FL	De-ID/Synth	Transport Realism	Governance
Meden et al. [16]	2021	✓	✓	✓	✓	✓	∼ ^a^	∼ ^e^
Laishram et al. [11]	2025	✓	✓	∼	✓	✓	✗ ^b^	∼ ^f^
Krivokuća Hahn & Marcel [14]	2023	∼	∼	✗	✗	✗	✗ ^c^	✗
Labati et al. [7]	2016	∼	✗	✗	✗	✗	✓ ^d^	✓ ^g^
Belen-Saglam et al. [18]	2025	∼ ^i^	✓	✗	✓	✓	✓ ^j^	✓ ^k^
This survey	2026	✓	✓	✓	✓	✓	✓	✓ ^h^

^a^ General biometrics; transport not primary. ^b^ Focus on PPFR mechanisms. ^c^ Template protection emphasis. ^d^ ABC constraints and usability. ^e^ Includes ISO/GDPR background; focus on B-PET technical effectiveness. ^f^ Lists international laws; analytical framework remains technical-algorithmic. ^g^ Section on legal compliance and ABC-specific privacy directives. ^h^ Core dimension (D3); links technical safeguards to necessity/proportionality. ^i^ Covers risks across data generation, storage, and sharing phases. ^j^ Specifically focused on Mobility-as-a-Service (MaaS) ecosystems. ^k^ Comprehensive focus on GDPR, CCPA, and organizational policies for transport.

**Table 2:** Overview of extracted analysis dimensions.

Dimension	Categories
Transport Domain	Airport, public transport, multi-domain
Use case	Access control, surveillance, crowd analytics, etc.
System Architecture	Centralized, edge, hybrid
PPFR	HE/MPC, FL, template protection, etc.
Privacy Leakage Focus	Identity, inference, linkage, membership
Datasets	Public, proprietary, transport-specific
Governance	None, implicit, explicit

**Table 3:** Counts used in Figure 5 (thematic focus by region).

Region	FRS	PPFR	Governance	Total
Asia-Pacific	24	10	4	38
Europe	14	15	22	51
North America	9	14	6	29
Middle East/Gulf	7	2	1	10
Latin America & Africa	4	1	9	14

**Table 4:** Transport-domain segmentation: operational constraints, PPFR fit, and main deployment gaps across the five categories identified in the corpus. Abbreviations; HE: homomorphic encryption; FL: federated learning; TEE: trusted execution environment; TP: template protection; De-ID: de-identification.

Domain	Key Operational Constraints	Applicable PPFR Techniques	Main Deployment Gap
ABC e-gates	Sub-second latency; strict 1:1 identity verification; ICAO compliance	TEE; lightweight HE	HE/MPC latency overhead exceeds gate budgets; no ICAO-certified PPFR architecture exists
Biometric boarding	Multi-actor data flows; cross-organization accountability; purpose limitation	FL; TP with revocable templates	Cross-actor responsibility undefined; data minimization rarely enforced across vendors
Metro/rail fare gates	High concurrency; motion blur; lighting variability; sub-second throughput	Edge FL; lightweight De-ID	No certification standard equivalent to ICAO; centralized storage dominates without template protection
Hub video analytics	Non-identifying processing; scalability; real-time crowd monitoring	De-ID; edge computing	No clear boundary between analytics and identification; function creep risk largely unmodeled
In-vehicle driver monitoring	Continuous worker monitoring; real-time processing; consent; power asymmetry	On-device FL; local inference	No sector-specific regulation; labor rights and governance frameworks largely absent

**Table 5:** Overview of dataset categories and evaluation practices in transport-related FRS studies.

Dataset Category	Typical Use	Limitations in Transport Contexts
General-purpose face datasets	Model training, pre-training, and baseline benchmarking	Controlled capture conditions; limited motion blur and occlusions; predominantly frontal poses; non-representative demographics
Transport-domain datasets	Evaluation under operational conditions (airports, metro, fare gates)	Proprietary access; restricted availability; limited reproducibility and cross-study comparison
Masked-face datasets	Robustness evaluation under face coverings	Narrow scope; task-specific; limited applicability beyond pandemic-related scenarios
Synthetic face datasets	Privacy-aware evaluation; bias and fairness analysis	Domain gap with real deployments; limited realism of transport environments

**Table 6:** High-level comparison across technical PPFR (C1), transport deployments (C2), and governance/socio-legal studies (C3) using the tri-dimensional framework (D1–D3).

Dimension	C1: Technical PPFR	C2: Transport Deployments	C3: Governance & Rights
Primary objective	Leakage reduction under formal threat models	Operational performance (FAR/FRR, latency, throughput)	Legitimacy, necessity, proportionality, accountability
System boundary	Isolated component (matching, training, template transform)	End-to-end gate/kiosk + backend pipeline	Institutional ecosystem (airport, airline, authority)
Strength (D1–D3)	Strong D1; weak D2–D3 unless modeled	Strong D2; partial D1; weak D3 reporting	Strong D3; weak D2; technical detail abstracted
Recurring blind spot	Retention, lawful basis, subgroup impacts	Privacy-by-design, threat models, fairness auditing	Latency, certification, implementation feasibility

**Table 7:** Tri-layer misalignment across technical PPFR, transport deployments, and governance literature.

Design Question	Technical PPFR Literature	Transport Deployments	Governance & Rights
Primary “risk”	Leakage or inference from templates, embeddings, gradients, or models [11,16,59,63].	Operational failure modes: throughput, false rejects, spoofing robustness [7,23,48].	Illegitimate surveillance: disproportionality, consent failure, discrimination, function creep [2,3,4,17].
System boundary	Often an isolated component; actors abstracted [36,49,61].	End-to-end gate/kiosk pipeline; multi-actor flows under-described [46,48].	Institutional ecosystem as unit of analysis [2,65].
Evidence of “success”	Accuracy plus security metric under stated threat model [12,32,66].	Queue stability, latency, usability, acceptance [7].	Compliance and legitimacy: necessity, transparency, redress, audits [17,51,62].
Typical blind spot	Governance (roles, lawful basis, retention) and subgroup impacts [52].	Privacy-by-design mechanisms and subgroup reporting [52].	Feasibility constraints (latency budgets, certification) [7,8].
Resulting misalignment	Strong privacy guarantees but weak deployment fit (latency, hardware, actors).	Operationally viable but privacy/governance opaque deployments.	Normatively robust requirements without consistent technical instantiation.

**Table 8:** Paper-level PPFR comparative matrix following the L1–L6 leakage taxonomy. Symbols: ✓ (full support), ∼ (partial support), ✗ (no support).

Year	Reference	Technique	L1	L2	L3	L4	L5	L6	Transport Alignment Note
2005	Newton et al. [43]	De-identification (*k*-Same)	✓	∼	✗	✗	✗	✗	Best fit for CCTV analytics where individual identification is not required.
2011	Sadeghi et al. [71]	Encrypted matching (HE/GC)	✗	✗	✓	∼	∼	∼	Strong confidentiality; typically exceeds high-throughput gate budgets without acceleration.
2017	McMahan et al. [36]	Federated Learning (FedAvg)	∼	✓	✗	✓	∼	∼	Edge data retention aligns with airport/station silos; reduces central exposure.
2019	Tramer & Boneh [66]	Private inference (TEEs)	✗	∼	∼	✓	∼	∼	Pragmatic for biometric gates; requires side-channel and supply-chain governance.
2024	Lei et al. [15]	Airport De-identification	✓	∼	✗	✗	✗	✗	Grounded in airport CCTV equipment; illustrates de-ID for real-time security tracking.
2024	Wang et al. [89]	Multi-key HE (PUM)	✗	✗	✓	✗	✓	∼	Mitigates template leakage in campus/cloud scenarios via feature grouping.
2025	Barman et al. [90]	FedBioAuth (FL + DP)	∼	✓	∼	✓	∼	✗	Specifically designed for multi-authority collaboration (Air, Rail, Border).
2026	Wang et al. [57]	FrQEM watermarking	✗	✗	✓	✗	✗	✗	L3 integrity verification for deepfake detection; robustness limited to conventional attacks.
2026	Wang et al. [58]	Diffusion watermark removal (WARDM)	✗	✗	✓	✗	✗	✗	Adversarial removal across orthogonal moment and deep learning watermarks; relevant to L3 threat modeling.

**Table 9:** Paper-level deployment matrix for biometric and surveillance technologies in transport infrastructures. Leakage columns (L1–L6) indicate exposed artifacts in described architectures (not mitigations). Governance column indicates whether concrete governance information is reported (retention, legal basis, opt-out, audits). Symbols: ✓ (full support), ∼ (partial support), ✗ (no support).

Year	Reference	Architecture	Primary Metric	L1	L2	L3	L4	L5	L6	Governance Info (Examples)
2012	Spreeuwers et al. [23]	e-Gates (Schiphol)	FRR/FAR, throughput	✓	∼	✓	✗	✓	∼	Analysis of deficiencies in digital passport photo quality.
2015	Frontex [53]	ABC systems (EU/Schengen)	FAR/FRR targets	✓	✓	✓	∼	∼	✓	Technical best practices and comprehensive data protection guidelines.
2016	Labati et al. [7]	Multi-modal e-Gates	Accuracy/ Throughput	✓	✓	✓	✓	✓	✓	Detailed analysis of legal frameworks, ethics, and ISO standards.
2021	Kasim et al. [31]	Extended TPB model (SEM)	Intention ($R^{2}$)	✓	✓	∼	✗	✗	✓	Privacy concerns identified as a significant moderator of passenger behavior.
2021	NASEM [46]	Airport ecosystem	Operational guidance	✓	✓	✓	✓	✗	✓	Comprehensive overview of policies, privacy planning, and IT architecture.
2022	Kumar et al. [29]	IoT + Deep CNN	97% accuracy	✓	✗	✓	✗	✗	∼	Real-time alert notifications for public health rule compliance.
2022	Guerrieri & Parla [30]	YOLOv3 edge analytics	Precision/Recall	✗	✗	✓	✗	✗	∼	Monitoring of social distancing and mask use in transit.
2023	Efatinasab et al. [37]	RF/RNN (CAN Bus)	Accuracy/ASR	✓	✓	✓	✓	✓	✓	Local training requirements ensure biometric data never leaves the vehicle.
2023	Sung [48]	Intelligent Border Management System (Korea)	Match scores	✓	✓	✓	✗	∼	✓	Socio-technical critique of surveillance racism and algorithmic profiling.
2025	Srivastava [92]	YOLO + federated learning	Inference speed	✗	✗	✓	✓	✗	✓	Privacy-by-design framework using decentralized training and encryption.

**Table 10:** Governance evidence used in Section 6. Sources provide operational criteria (necessity, transparency, accountability) that map to design and reporting practices.

Reference	Type	Transport Focus	Governance Criteria	Design Implications
GDPR [51]	Regulation	Special-category biometric processing in transport	Lawful basis; minimization; purpose limitation; DPIA; data-subject rights	Constrains system design across layers: retention, accountability, lawful processing.
CNIL guidance [62]	DPA guidance	Biometrics in public-facing contexts	Necessity/proportionality; transparency; alternatives; retention; vendor roles	Defines operational safeguards often absent in deployment studies.
EDPB opinion [2]	DPA board opinion	Facial recognition in public spaces	High-risk uses; strict necessity; safeguards; bans/limitations	Adds proportionality as explicit evaluation dimension beyond leakage.
EU AI Act [1]	Regulation	High-risk biometric identification systems	Risk management; documentation; oversight; robustness; monitoring	Expands evaluation beyond accuracy to documentation and post-market duties.
Fussey et al. [4]	Independent analysis	Live FR in public space (terminals/CCTV)	Legitimacy; transparency; bias; operational drift	Introduces “surveillance creep” rarely modeled in PPFR threat analysis.
Lyon [3]	Surveillance studies	Normalization in mobility infrastructures	Chilling effects; power asymmetry; secondary use	Supports claim that legality and trust exceed leakage metrics.
Negri & Silva [17]	Legal scholarship	Proportionality tests for public biometrics	Structured necessity tests; safeguards; accountability	Maps directly to governance dimension: technical controls necessary but insufficient.
[65,93]	Socio-technical analysis	Airport biometrics as multi-actor system	System boundaries; shared responsibility; assurance evidence	Justifies governance as a design parameter in PPFR evaluation.

## Data Availability

No new data were created or analyzed in this study. Data sharing is not applicable to this article.

## Abbreviations

The following abbreviations are used in this manuscript:

FR	Face Recognition
FRS	Face Recognition Systems
FRT	Face Recognition Technology
ABC	Automated Border Control
PPFR	Privacy-Preserving Face Recognition
SLR	Systematic Literature Review
HE	Homomorphic Encryption
FRVT	Face Recognition Vendor Test
FAR	False Acceptance Rate
FRR	False Rejection Rate
EER	Equal Error Rate
FL	Federated Learning
